# Frontiers Commentary: The HEART Mobile Phone Trial: The Partial Mediating Effects of Self-Efficacy on Physical Activity among Cardiac Patients

**DOI:** 10.3389/fpubh.2016.00066

**Published:** 2016-04-15

**Authors:** Dan J. Graham

**Affiliations:** ^1^Department of Psychology, Colorado School of Public Health, Colorado State University, Fort Collins, CO, USA

**Keywords:** mHealth, mobile phone, smartphone, physical activity, self-efficacy

Maddison and colleagues’ HEART mobile phone trial (1) presents dual insights that will be useful and important to those promoting physical activity (PA) and other health behaviors in the twenty-first century. First, interventions delivered by smartphone can increase PA among a clinically important population (individuals with heart disease). Second, increasing self-efficacy is an important mechanism for mobile health (mHealth) interventions to target.

As has been demonstrated previously, self-efficacy is among PAs most reliable correlates and even determinants (2, 3). This robust relationship between self-efficacy and PA exists for children and adolescents (4–6) as well as younger and older adults (7–9). Furthermore, much intervention research has shown that successfully increasing PA self-efficacy leads to increased PA in both healthy and obese populations (10–12). Self-efficacy’s enhancement of PA has been demonstrated via interventions delivered in-person (13), by print-based materials (14), by telephone (15), by mass media (16), by postal mail (14), and by combinations of these (17, 18). Email has also been used to deliver PA intervention content with some success (19), and with smartphones representing one of the most rapidly adopted technologies ever documented (20), there is reason to believe that email and other smartphone-mediated delivery will be at least as successful in years to come. Although the HEART trial reveals the importance of increasing self-efficacy in the context of promoting PA (1), the principle is likely more broadly applicable. This evidence that self-efficacy can be effectively increased in mHealth interventions should be encouraging to public health practitioners aiming to improve not only PA but also other health behaviors that have been positively impacted by self-efficacy change in other intervention contexts. Reducing smoking and drinking, increasing contraceptive use, and healthy eating all have long established (21) as well as consistently and recently reaffirmed ties to increased self-efficacy (22–25).

Maddison and coauthors indicate that future mHealth research promoting PA would benefit from incorporating objective activity assessment (1); this is an increasingly feasible goal, even with large-scale interventions. Smartphone-mediated interventions can permit accelerometers already present in smartphones, and already capable of assessing PA (26), to be linked with intervention apps, so that content could be tailored based on users’ activity. For example, if a phone’s accelerometer has not recorded some designated level of movement over the past two daytime hours, the user could receive a text message suggesting a brief walk. Such an alerting feature is available in some commercial activity monitors (e.g., Garmin vivofit, Jawbone UP) and could comprise a useful element of mHealth PA interventions. Additionally, interventions could incorporate persuasive technologies (27) like those used in home energy meters that glow one color when energy use is low and a different color when energy use is high. Similarly, smartphone users can benefit from simple visible cues to modify their own energy expenditure (28). Smartphones are already being used to assess PA speed and location (29), and as technology continues to advance, current obstacles to data quality such as sensor disruptions due to competing power demands or the phone being worn/carried in different positions (30) will likely diminish.

Maddison and colleagues provide encouraging evidence of mHealth success even in the absence of substantial tailoring of intervention content (1). The ability to tailor content, a strategy previously shown to be successful in increasing self-efficacy and PA (31) is enhanced substantially in mHealth interventions. It is reasonable to assume that tailored intervention content delivered to smartphones would improve intervention outcomes as tailoring has done through print delivery channels (32). Indeed, meta-analytic results identify tailoring based on self-efficacy as a particularly promising strategy (32). Moving forward, mHealth interventions may be even more successful if they employ active assistance technology (33) that is not only tailored, but adaptive (i.e., interacting with the user in an ongoing way, not merely utilizing one-time, *a priori* tailoring).

Online and smartphone-based games are also very popular, being played by nearly half of all Internet users (34). Gaming represents another excellent electronic venue through which interventionists can promote health behavior change [e.g., Ref. (35, 36)]. Some health-promotion video games have produced beneficial effects by increasing self-efficacy for important health behaviors [e.g., Ref. (37, 38)]. As video game technology provides increasingly immersive experiences, interventions incorporating gaming may well be even more effective in increasing player self-efficacy and health behaviors (39). Virtual reality technology could further enhance self-efficacy (e.g., by seeing one’s virtual self engaging in efficacious health-promotion acts) (40).

The greatest potential barrier to mHealth interventions, the inability to access individuals who lack necessary electronic devices, is rapidly reducing. With over 50% of the populations of nearly 20 countries owning smartphones as of 2013 (41), and projections that over half of the world’s 4+ billion mobile phone users will have smartphones by 2016 (42), many more individuals will soon be able to access smartphone-mediated interventions. In several countries (e.g., South Korea, Australia, Israel, United States, Spain), smartphone ownership currently exceeds 70% (41). In the US, 64% of all Americans, and 85% of young adults, currently own smartphones, and these rates are rapidly increasing (e.g., only 35% of US adults owned smartphones 4 years ago) (43). In addition, mHealth interventions may not necessarily exclude those most in need of targeted efforts. Public health practitioners previously hampered by the inability to access those living in rural areas, low-income areas, and minority communities (44) may actually find mHealth programs well-suited to reaching racial and ethnic minority groups that have traditionally faced greater health inequities (45). For example, rates of smartphone ownership are higher among Hispanic and Black Americans than among White Americans (43).

The success of the HEART mobile phone trial provides promising strategies for researchers and public health practitioners to adopt and expand upon. Future mHealth interventions may benefit from incorporating location- and/or movement-based content delivery, message tailoring, persuasive and active assistance technologies, video games, and dissemination to a variety of groups including at-risk populations. Such strategies have great potential to enhance public health in an affordable and far-reaching manner.

## Author Contributions

The author confirms being the sole contributor of this work and approved it for publication.

## Conflict of Interest Statement

The author declares that the research was conducted in the absence of any commercial or financial relationships that could be construed as a potential conflict of interest.
